# Supplementary material for: A Global Screen for Assembly State Changes of the Mitotic Proteome by SEC-SWATH-MS
Source: Cell Syst. 2020 Feb 26;10(2):133–155.e6. doi: 10.1016/j.cels.2020.01.001 (PMC7042714; doi:10.1016/j.cels.2020.01.001)

P28290 | SSFA2_HUMAN | SSFA2 CS1 KIAA1927 KRAP
Monomer MW [kDa]: 138.386 Monomer expected elution fraction: 37

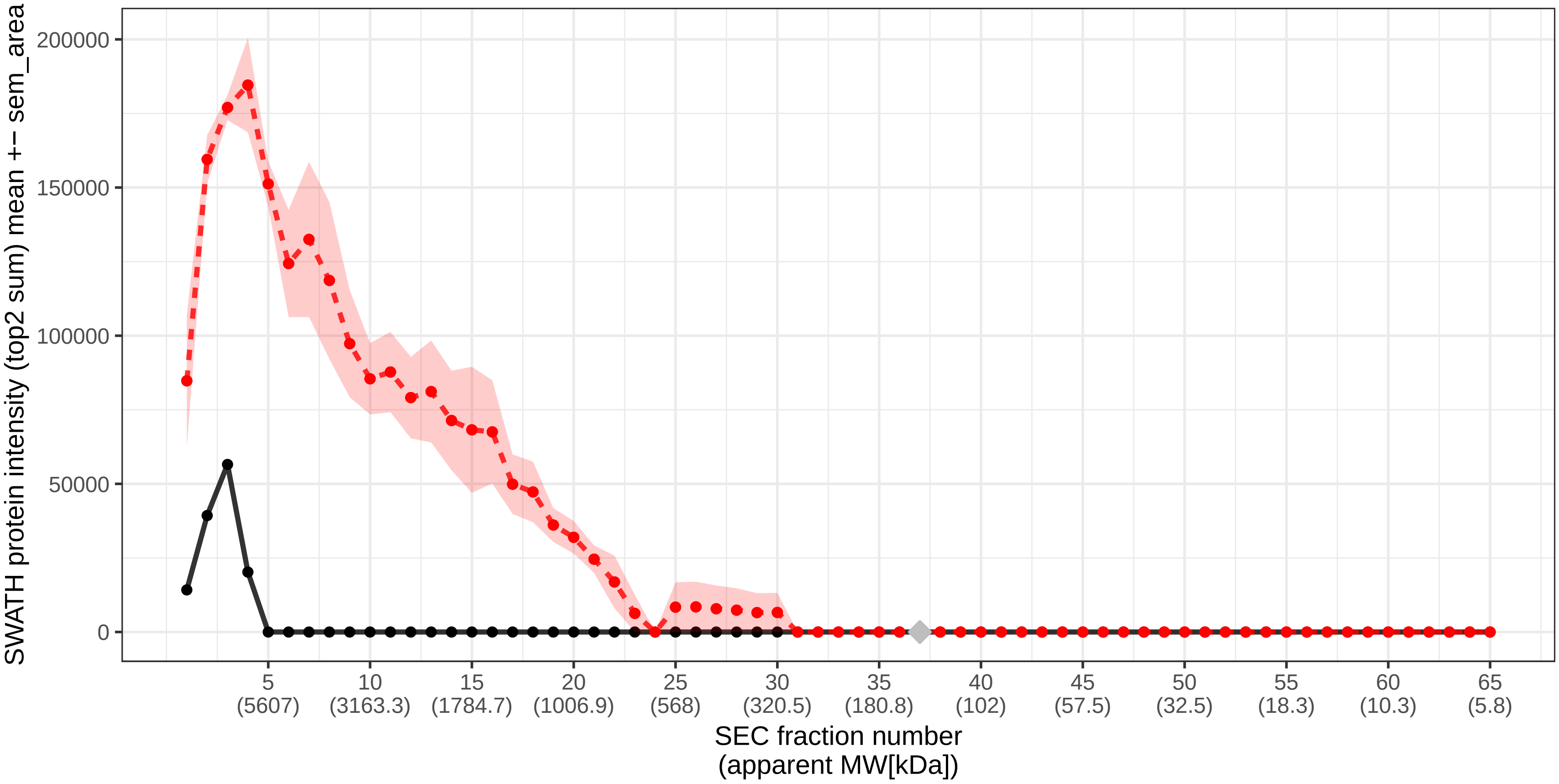

Supplement: Data S1. SEC-SWATH-MS Protein Chromatograms, Related to Figure 1 [file mmc6.zip › SECchrom_P28290_SSFA2_HUMAN_SSFA2_CS1_KIAA1927_KRAP.pdf]
